# Determinants of Rabies Post-Exposure Prophylaxis Compliance in Bangladesh: Informing Policy for Elimination by 2030

**DOI:** 10.3390/tropicalmed11060165

**Published:** 2026-06-18

**Authors:** Sumon Ghosh, Mohammad Nayeem Hasan, Sukanta Chowdhury, Narayan C. Paul, Waqas Ahmad, Jiangang Chen, Thankam S. Sunil

**Affiliations:** 1 Department of Nutrition and Public Health Sciences, College of Education, Health, & Human Sciences, University of Tennessee, 382 HPER, 1914 Andy Holt Ave., Knoxville, TN 37996, USA; jchen38@utk.edu (J.C.); tsunil@utk.edu (T.S.S.); 2 Monitoring, Evaluation & Research Department, Community Partners International, East Dolphin Point, Cox’s Bazar 4700, Bangladesh; nayeem5847@gmail.com; 3 International Centre for Diarrhoeal Disease Research (icddr,b), Dhaka 1212, Bangladesh; sukanta@icddrb.org; 4 Breathitt Veterinary Center, Hutson School of Agriculture, Murray State University, Hopkinsville, KY 42240, USA; npaul3@murraystate.edu; 5 Department of Clinical Sciences, University of Veterinary and Animal Sciences, Lahore, Narowal Campus, Narowal 51600, Pakistan; waqas.hussain@uvas.edu.pk

**Keywords:** animal bite, vaccine adherence, rabies vaccines, health-seeking behavior, One Health, rabies elimination

## Abstract

Rabies remains a preventable yet fatal zoonotic disease and a major public health concern in Bangladesh, which aims to eliminate dog-mediated human rabies by 2030. Despite free availability of post-exposure prophylaxis (PEP), adherence to the WHO-recommended PEP regimen remains low. This study assessed PEP compliance and identified determinants of regimen completion among animal-exposed patients. We conducted a hospital-based observational study using secondary data from 457 patients who initiated PEP at the National Rabies Prevention and Control Centre (NRPCC) in Dhaka, from February 2023 to July 2023. Sociodemographic, clinical, and exposure-related factors were analyzed to identify predictors of compliance. Only 17.1% of patients completed the full PEP regimen, including rabies immunoglobulin (RIG) administration for WHO Category III exposures where indicated. Higher adherence was observed among females, individuals aged ≥15 years, lower-income groups, and those residing within 10 km of the treatment center. Exposure-related factors including dog bites, multiple exposures, unprovoked incidents, and appropriate exposure care were also associated with improved compliance. Despite free access, PEP completion remains critically low. Targeted strategies, including decentralized PEP delivery, improved public awareness, and strengthened follow-up systems, are essential to improve adherence and support progress toward rabies elimination by 2030.

## 1. Introduction

Rabies is a fatal but preventable zoonotic viral disease that can cause approximately 59,000 human deaths worldwide annually, predominantly in Asia and Africa, where canine rabies remains endemic [1,2]. Dogs account for approximately 99% of human rabies cases, making control of dog-mediated transmission a crucial public health priority [3,4]. Despite the existence of safe and effective vaccines for over a century, rabies continues to cause preventable deaths, particularly in low- and middle-income countries where gaps in awareness, healthcare access, and program implementation persist [5]. Post-exposure prophylaxis (PEP), consisting of thorough wound cleansing, administration of a rabies vaccine, and, for category III exposures, rabies immunoglobulin (RIG) [6], is the only proven intervention to prevent disease onset following potential rabies exposure [7].

In Bangladesh, rabies constitutes a significant public health threat, with an estimated 2100 human deaths annually, placing the country among those with the highest rabies mortality rates globally [8,9,10]. Bangladesh has committed to eliminating dog-mediated human rabies by 2030, aligned with the WHO-led “Zero by 30” initiative through sustained mass dog vaccination (MDV), multi-sectoral public education, and expanded access to free PEP [4]. Nevertheless, achieving this goal requires ensuring high compliance with PEP among individuals exposed to potentially rabid animals [11]. Earlier reports from Bangladesh have documented encouraging levels of PEP uptake, yet substantial proportions of patients fail to complete the full WHO-recommended PEP regimen, including completion of the vaccine schedule and receipt of RIG for Category III exposures where indicated [12,13]. Factors contributing to non-compliance include poor awareness of rabies and PEP schedules, financial hardship, long travel distances, difficulty accessing clinics due to work commitments, and the inconvenience of multiple follow-up visits [14]. Identifying and addressing these barriers is critical for ensuring the effectiveness of national rabies elimination efforts. Bangladesh provides free PEP at designated centers, following WHO guidelines for intradermal vaccine administration on Days 0, 3, and 7, with RIG given for Category III exposures [15,16].

Despite significant investments in rabies control in Bangladesh, including free PEP services and nationwide rabies elimination efforts, there is limited evidence of completion of the full WHO-recommended PEP regimen among animal-exposed patients at the national referral level. Most previous studies in Bangladesh have primarily focused on rabies epidemiology, awareness, or initial healthcare-seeking behavior, with comparatively little attention given to determinants of PEP completion and adherence behavior. In addition, evidence on how sociodemographic characteristics, exposure severity, healthcare accessibility, and behavioral responses collectively influence compliance remains insufficient in the Bangladesh context. Addressing these knowledge gaps is critical to identify vulnerable groups at risk of poor adherence to the WHO-recommended PEP regimen and to develop targeted interventions that support rabies prevention and elimination efforts. Evidence remains limited on the true rate of compliance with the full WHO-recommended PEP regimen and the factors associated with incomplete adherence in the Bangladesh context. Therefore, this study aimed to determine the rate of compliance with the WHO-recommended rabies PEP regimen among animal-exposure patients in Bangladesh and to identify key sociodemographic, clinical, and contextual factors associated with non-compliance. Insights from this study could guide targeted interventions to improve adherence and support Bangladesh’s progress toward rabies elimination.

## 2. Methods

We obtained immunization records of 457 patients from the National Rabies Prevention and Control Centre (NRPCC), located within the Infectious Diseases Hospital (IDH) in Dhaka, Bangladesh. NRPCC serves as Bangladesh’s primary referral center for animal-exposed patients and rabies cases, providing free WHO-recommended rabies PEP, including vaccination and RIG when indicated, to patients nationwide [5]. The study included patients of any age or sex who presented to the NRPCC and initiated WHO-recommended rabies PEP between February and July 2023. Because the study was based on routinely collected secondary immunization records from a government referral center, all eligible records during the study period were included, while records with missing or incomplete information on PEP completion or key study variables were excluded from the analysis. We examined a comprehensive set of socioeconomic and clinical variables as independent predictors to identify potential risk factors for failure to complete the recommended PEP regimen. These included age, sex, occupation, income level, highest educational attainment, residential location, and distance to NRPCC, prior awareness of the rabies treatment center, details of the animal exposure (species, nature of exposure, number of exposures, exposure category, biting animal status, location, and reason for the exposures), exposure-care measures taken and reported reasons for delayed attendance.

### 2.1. Variables and Categorization

The primary dependent variable was compliance with the WHO-recommended rabies PEP regimen used at the NRPCC during the study period, which included intradermal rabies vaccine administration on Days 0, 3, and 7, with RIG administered for Category III exposures. Patients who completed all recommended vaccine doses and, where indicated, received RIG for Category III exposures were classified as compliant, whereas those who failed to complete the recommended PEP regimen were considered non-compliant. The independent variables included a range of sociodemographic, clinical, and exposure-related factors. Age was categorized into two groups: <15 years and ≥15 years. Sex was coded as male or female. Occupation was classified as student, job/business, housewife, child, or other. Monthly household income was dichotomized as < BDT 30,000 (USD 1 ≈ BDT 122) and ≥BDT 30,000 (USD 1 ≈ BDT 122). Educational attainment was grouped into five categories: no education or below primary, primary, secondary, higher secondary, and above higher secondary education. Residential distance from the treatment center (NRPCC) was categorized as <10 km or ≥10 km based on the sample means. Prior knowledge of the IDH was recorded as a binary variable (yes/no), and the source of information about the facility was classified as doctor, neighbor, relative, or other.

Exposure-related variables included animal type (dog, cat, or other), recorded exposure type (bite or scratch or mucosal lick), number of exposure events (single or multiple), animal ownership status (stray, community-owned, own pet, or wild), and circumstances of exposure (provoked by patient, provoked by animal, or unprovoked). Exposure severity was further classified according to WHO criteria as Category I, II, or III. Exposure-care practices following the exposure were grouped as washing with water and soap, washing with water only, no treatment, or other measures.

All variables were coded appropriately for statistical analysis, and categories were selected based on their relevance in prior literature and epidemiological plausibility.

**Figure 1:**
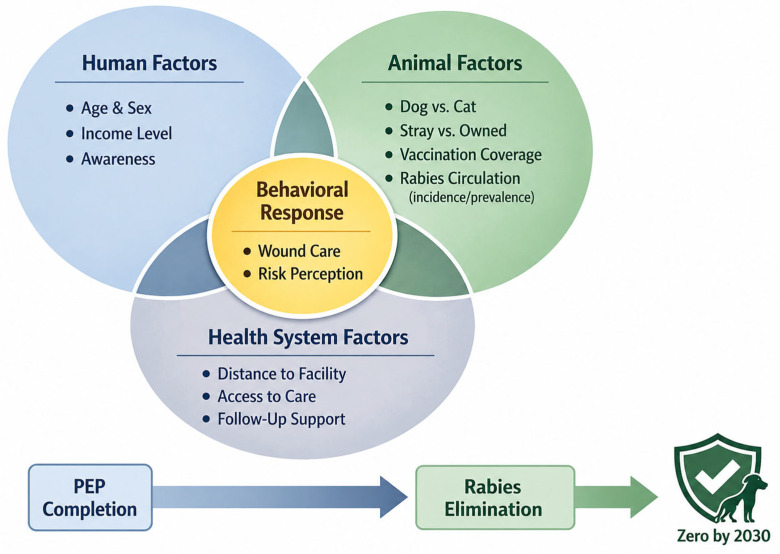
One Health conceptual framework illustrating how human, animal, and health-system factors interact through behavioral responses to influence rabies post-exposure prophylaxis (PEP) completion and progress toward dog-mediated rabies elimination in Bangladesh (“Zero by 2030”).

This study was informed by a One Health conceptual framework integrating human, animal, and health-system determinants of rabies PEP compliance. Sociodemographic characteristics, exposure-related factors, and healthcare access variables were hypothesized to interact through behavioral responses—particularly wound care practices and risk perception—to influence PEP completion and progress towards rabies elimination (Figure 1).

### 2.2. Statistical Analysis

We summarized categorical variables using contingency tables (counts and percentages) and applied Pearson’s chi-square test when expected cell counts were adequate, while Fisher’s exact test was used for tables with small expected frequencies (i.e., when >20% of cells had expected counts < 5 or any cell < 1), to ensure the validity of statistical testing under the respective test assumptions [17]. We then employed logistic regression to identify factors associated with PEP compliance. Initially, univariable (crude) logistic regressions were carried out for individual predictors, with variables showing *p* < 0.20 considered for multivariable modeling—a common strategy in epidemiology to avoid premature exclusion of important predictors [18]. The multivariable model included all selected variables simultaneously, and results are reported as odds ratios (OR) with 95% confidence intervals, using a two-sided significance threshold of *p* < 0.05 [19]. Multicollinearity was assessed via variance inflation factors (VIF), with values below 4 retained in the model [20]. All analyses were performed in IBM SPSS version 25.0, and the map was produced by R version 4.5.0.

### 2.3. Model Performance

We evaluated the predictive performance of the final logistic regression model using several complementary measures. Discrimination was assessed via the area under the receiver operating characteristic curve (AUROC), which quantifies how well the model distinguishes between compliant and non-compliant patients: values closer to 1 indicate stronger discrimination, with ≥0.70 considered acceptable [21]. Sensitivity and specificity were also derived at the optimal threshold to further characterize model accuracy. Overall goodness-of-fit was tested using the Hosmer–Lemeshow statistic: a non-significant *p*-value (>0.05) implies that predicted probabilities align well with observed outcomes [22].

## 3. Results

**Figure 2:**
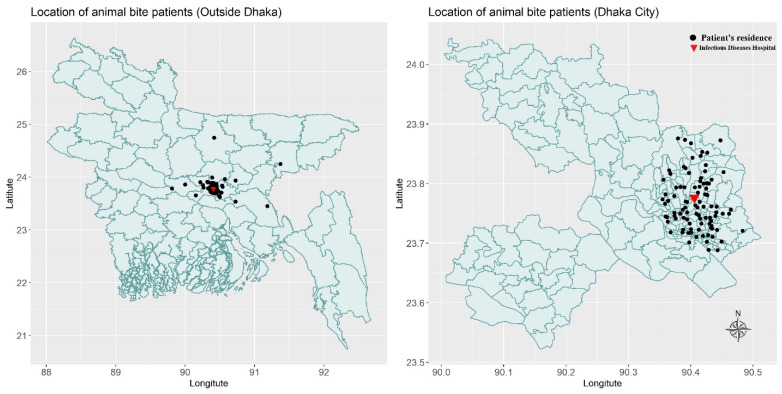
Map showing the geographic distribution of animal-exposure cases who reported to the National Rabies Prevention and Control Centre at the IDH, Dhaka, Bangladesh (February–July 2023).

Between February and July 2023, data from 457 rabies vaccine recipients at the NRPCC were extracted from immunization records for a secondary analysis of patient demographics, exposure characteristics, and PEP adherence, representing a subset of all individuals who attended the center during that period. The majority of animal exposure cases who reported to the NRPCC were clustered in Dhaka city and its immediate surroundings, with several patients coming from peripheral districts outside the metropolitan area (Figure 2). The mean age of patients presenting with animal exposures was 25.9 years (SD = 15.6) (Table 1). The average monthly household income was BDT 34,105 (USD 1 ≈ BDT 122), with notable variability (SD = 45,119). Participants resided an average distance of 10.4 km (SD = 9.4) from the IDH, incurring mean travel costs of BDT 148 (SD = 113) (USD 1 ≈ BDT 122). The interval between exposure and presentation at the hospital averaged 3.2 days (SD = 14.8), although delays varied widely among patients. Medication expenses showed relatively little variation, averaging BDT 345 (SD = 46) (USD 1 ≈ BDT 122).

**Figure 3:**
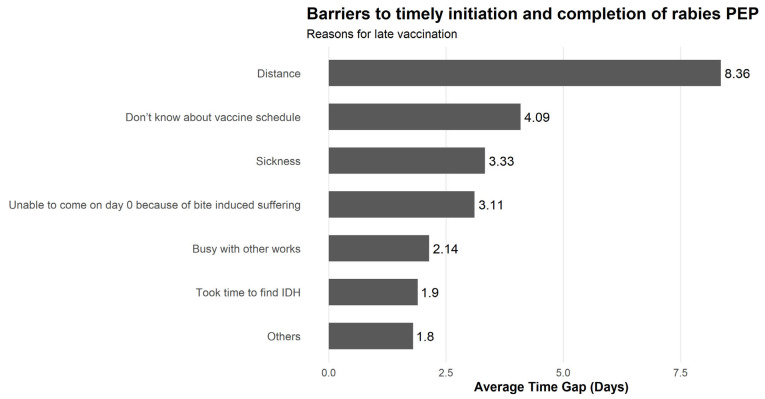
Bar chart illustrating mean delays (in days) for delayed attendance to rabies post-exposure prophylaxis (PEP) at the National Rabies Prevention and Control Centre, IDH, Dhaka, Bangladesh (February–July 2023).

Among patients reporting delays in completing the PEP regimen, distance to the healthcare facility contributed to the greatest mean delay (8.36 days), followed by lack of awareness of the PEP regimen (4.09 days) (Figure 3). Other contributing factors included illness (3.33 days), exposure-related complications (3.11 days), work obligations (2.14 days), difficulty locating the hospital (1.90 days), and miscellaneous reasons (1.80 days).

Of the 457 patients included in the study, 78 (17.1%) completed the full WHO-recommended rabies PEP regimen. Individuals aged ≥15 years had higher adherence than those under 15 years (19.1% vs. 11.5%, *p* = 0.055), and females were more compliant than males (22.5% vs. 14.6%, *p* = 0.037) (Table 2). Patients who received RIG showed significantly higher PEP compliance than those who did not receive RIG (21.3% vs. 12.4%, *p* = 0.013). Profession was also linked to compliance (*p* = 0.027), with housewives (23.2%) and the “other” occupation group (30.0%) outperforming students and business workers. Lower-income participants (<BDT 30,000) (USD 1 ≈ BDT 122) demonstrated markedly better adherence than their higher-income counterparts (28.4% vs. 7.6%, *p* < 0.001). Although not statistically significant (*p* = 0.114), compliance was slightly higher among those with no formal or primary education.

**Figure 4:**
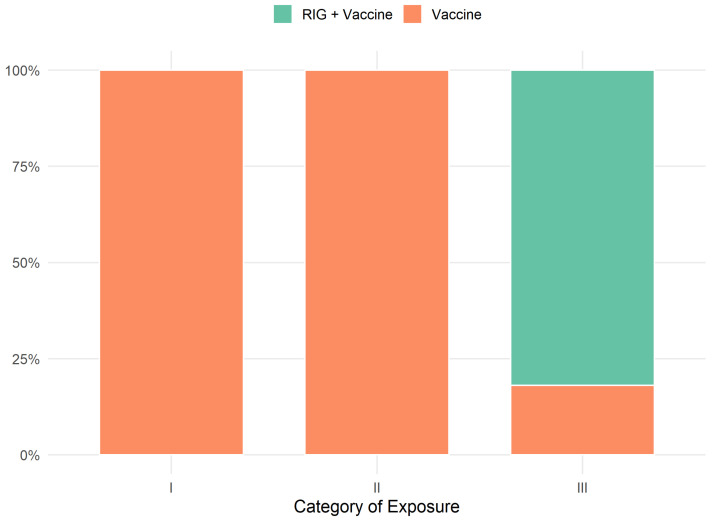
Distribution of rabies immunoglobulin (RIG) and anti-rabies vaccine (ARV) administration according to WHO exposure categories among patients with animal exposures who reported to the National Rabies Prevention and Control Centre (NRPCC), Dhaka, Bangladesh, 2023.

Residing within 10 km of the hospital increased adherence (22.1% vs. 11.1%, *p* = 0.002). Notably, those unfamiliar with the hospital before their visit had higher compliance (28.1% vs. 13.4%, *p* < 0.001), and those informed about the facility by relatives or others were more compliant than those informed by doctors or neighbors (*p* < 0.001). In terms of exposure, dog-associated exposures (21.9%) were associated with higher compliance than cat or other animal-associated exposures (*p* = 0.020), and bite wounds elicited better adherence than scratches (21.1% vs. 11.5%, *p* = 0.008). Multiple exposures (23.0%) improved compliance relative to single exposures (14.8%, *p* = 0.037), and exposure to stray animals showed a trend toward higher adherence (*p* = 0.055). Unprovoked exposures (22.5%, *p* = 0.004) and WHO Category III exposures (17.4%, *p* = 0.007) were associated with higher compliance. Patients who washed the wound with water only (AOR 0.29; 95% CI 0.14–0.91; *p* = 0.024) or adopted other immediate protective measures (AOR 0.22; 95% CI 0.12–0.84; *p* = 0.022) were more likely to complete the recommended PEP series than those who took no action following exposure. Although washing with soap and water showed a similar trend, the association was not statistically significant after adjustment (AOR 0.48; 95% CI 0.17–1.98; *p* = 0.248). After multivariable adjustment, several factors remained independently associated with completion of the WHO-recommended rabies PEP schedule. Notably, individuals from low-income households (<BDT 30,000) (USD 1 ≈ BDT 122) had more than four times higher odds of completing the regimen compared with those with higher income (AOR 4.18; 95% CI 2.07–8.47; *p* < 0.001). Patients who received RIG showed significantly better compliance with the WHO-recommended PEP regimen in the crude analysis (COR 0.53; 95% CI 0.32–0.88; *p* = 0.013). RIG administration was limited to WHO Category III exposures, whereas most patients with Category II exposures received vaccines only; a small number of Category I individuals received vaccines inappropriately under WHO guidelines (Table 3, Figure 4). Female patients were significantly more adherent than males, with a 37% reduction in the odds of non-compliance (AOR 0.63; 95% CI 0.28–0.91; *p* = 0.025). Similarly, residing within 10 km of the hospital nearly quadrupled the odds of adherence (AOR 3.82; 95% CI 1.64–8.93; *p* = 0.002), while prior knowledge of the IDH reduced the odds of non-compliance by two-thirds (AOR 0.33; 95% CI 0.17–0.65; *p* < 0.001). In exposure-related factors, those experiencing scratches alone were more likely to adhere (AOR 1.77; 95% CI 1.16–3.66; *p* = 0.012), whereas patients with a single exposure had significantly lower odds of non-compliance compared with those with multiple exposures (AOR 0.41; 95% CI 0.20–0.81; *p* = 0.010). Exposure to stray animals (versus wild) also improved adherence (AOR 0.36; 95% CI 0.13–0.95; *p* = 0.039). Dog-associated exposures were associated with higher PEP compliance (AOR = 0.59; 95% CI: 0.25–0.94; *p* = 0.022). Among wound-care behaviors, patients who washed the wound with water only (AOR 0.29; 95% CI 0.14–0.91; *p* = 0.024) or undertook other post-exposure measures (AOR 0.22; 95% CI 0.12–0.84; *p* = 0.022) were more likely to complete the PEP regimen than those who did nothing following exposure.

**Figure 5:**
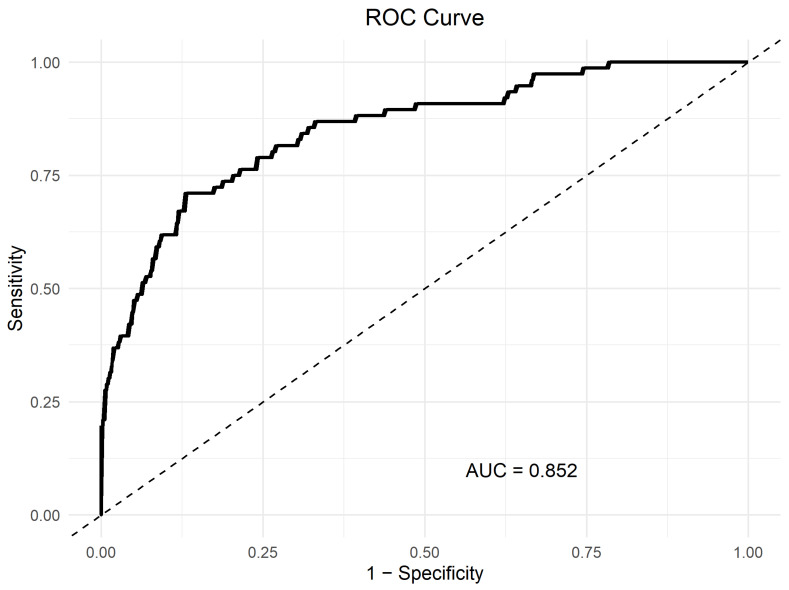
ROC curve of final multivariable logistic regression model.

The final logistic regression model demonstrated excellent performance: the Hosmer–Lemeshow test showed strong calibration (χ^2^ = 5.97, df = 8, *p* = 0.651), indicating no significant difference between observed and predicted outcomes (Table 4 and Figure 5). Discrimination was likewise high, with an area under the ROC curve (AUC) of 0.852 (95% CI: 0.840–0.875), reflecting excellent ability to distinguish between compliant and non-compliant patients (AUC > 0.80 is considered clinically useful). The model correctly classified 87.5% of cases, demonstrating strong predictive accuracy.

## 4. Discussion

This study provides important insights into rabies PEP compliance among patients with animal exposures in Bangladesh, revealing both encouraging and concerning trends. Our study revealed that adherence to the WHO-recommended rabies PEP remains low in Bangladesh, with merely 17.1% of patients with animal exposure completing the entire PEP regimen. Adherence was considerably higher among patients aged 15 years and older, as well as among females. Patients from low-income households were more likely to follow recommended PEP compliance than those from high-income groups. Proximity to the treatment center and not knowing about it before were also linked to better adherence. Factors associated with exposure, like dog exposure, unprovoked attackers, multiple exposures, and early wound care, were linked to better compliance.

We found the rate of adherence to the WHO-recommended PEP remains suboptimal, despite the availability of free PEP services nationwide. Our findings highlight several demographic, socioeconomic, and exposure-related factors that significantly influence patients’ likelihood of completing the full PEP regimen. These findings align with broader evidence from South Asia and Africa that highlights the multifactorial nature of PEP compliance, influenced by both accessibility and individual perceptions of risk [22,23]. Addressing these barriers is essential for optimizing individual patient outcomes and for supporting Bangladesh’s national “Zero by 30” rabies elimination goals.

The observed overall compliance in our study lines up with previous reports from Bangladesh and other endemic countries, where, although PEP uptake is generally high after initial visits, a significant proportion of patients do not follow the prescribed regimen [2,16,23,24,25]. Our analysis revealed that individuals from lower-income households were more likely to adhere to the PEP schedule compared with those with higher incomes. While this may appear counterintuitive, it could reflect differences in perceived disease risk, health-seeking behavior, and prioritization of free public health services among lower-income populations. Individuals from lower-income groups may have fewer alternative healthcare options or a greater perceived value of free public health services, and may prioritize completing the free PEP regimen once they access the facility. On the other hand, higher-income individuals might face different barriers, such as work obligations, which our study also identified as a contributing factor to delays. Additionally, some individuals may choose to receive subsequent vaccine doses from private facilities at their own expense after initiating PEP at a public hospital, thereby reducing the likelihood of documentation of their PEP record in public records. This contrasts with some studies that suggest that higher socioeconomic status is generally associated with better health-seeking behaviors and access to care [26,27]. The finding that lower-income individuals exhibit greater compliance when PEP is provided at no cost highlights the importance of sustained public funding for rabies prophylaxis as a vital strategy for achieving the national goal of eliminating dog-mediated human rabies by 2030 [28]. It also underscores the importance of equitable access to essential health services, particularly for diseases that disproportionately affect vulnerable populations in low- and middle-income countries [27].

We also found that gender differences in compliance were significant, where female patients showed higher adherence than their male counterparts, which is consistent with previous research indicating that, in South Asia, women often exhibit greater attention to health-related guidelines and are more actively involved in household health decisions [16,27,29]. However, this contrasts with broader gender norms in the region, where women may face reduced mobility or reliance on family decision-making for healthcare access. These conflicting influences—women’s heightened health vigilance versus cultural restrictions on their autonomy—highlight the necessity for additional qualitative research to elucidate the unique effects of gender roles on PEP compliance in Bangladesh.

Geographic proximity to the treatment center emerged as a critical determinant of adherence. Patients residing within 10 km of the hospital were significantly more likely to complete PEP, underscoring the challenge posed by travel distance and associated costs, even in settings where treatment itself is free. Similar barriers related to geographical access have been documented in rabies-endemic regions worldwide [30,31]. Innovative solutions, including decentralizing PEP services and integrating rabies management into primary healthcare facilities may help mitigate these challenges. Moreover, targeted awareness campaigns that emphasize the importance of completing the full PEP schedule, particularly for those living far from health facilities or those with higher incomes who might perceive fewer personal risks, are crucial. Educational initiatives should specifically address the common reasons for non-compliance, such as forgetting vaccine dates and work obligations, potentially through reminder systems like telephone calls or SMS messages, which have shown positive impacts on adherence [10].

Additionally, being unfamiliar with the treatment centre (NRPCC) prior to the visit and being informed about the facility by relatives or others were associated with higher compliance, suggesting that informal networks and the initial perceived novelty or criticality of the situation might enhance adherence [24]. Further qualitative research is necessary to explore these behavioral dynamics.

We also found exposure characteristics significantly influenced PEP compliance: patients with dog exposure, multiple exposures, and unprovoked attacks showed higher adherence to the PEP regimen, likely due to heightened awareness of rabies risk. Similar findings have also been documented with studies from both Asia and Africa linking Category III exposures to better compliance [24,32]. On the other hand, we found individuals presenting with scratches were less likely to complete the PEP regimen, possibly reflecting lower perceived rabies risk associated with these exposures, a factor previously linked to non-compliance. These findings highlight the significance of targeted educational campaigns, stressing that even seemingly minor exposures require full PEP, complying with public health guidelines [33]. WHO Category III exposures were also associated with increased PEP compliance, suggesting that patients with higher-risk exposures were more likely to complete the recommended regimen [14,34]. Notably, a small number of individuals classified as having WHO Category I exposures received rabies vaccine unnecessarily in routine clinical practice, despite PEP not being indicated for this category under WHO guidelines. This finding highlights the need for consistent exposure assessment and continued provider education to ensure adherence to recommended rabies management protocols. Patients who undertook immediate wound-care measures following animal exposure were generally more likely to complete the rabies PEP regimen than those who took no action. In the adjusted analysis, washing with water only and undertaking other measures following exposure remained significantly associated with improved adherence, whereas washing with soap and water showed a similar but non-significant trend. These findings emphasize the importance of promoting immediate wound care and public awareness to improve completion of the full PEP regimen [35,36].

For clinical practice, the study reinforces the need for clinicians to provide comprehensive counseling at the initial visit, emphasizing the importance of full adherence to the WHO-recommended PEP regimen and the potential consequences of non-compliance, particularly among patients who may underestimate the risk associated with WHO Category II and III exposures, including scratches and mucosal exposures [28]. Enhanced communication strategies, perhaps leveraging community health workers or digital reminders, are necessary to bridge the gap in awareness and counteract the effect of vaccine fatigue, particularly for later doses [10].

Although improving PEP adherence is essential for preventing human rabies deaths, sustainable elimination of dog-mediated rabies ultimately depends on effective control of the animal reservoir through sustained MDV and strengthened veterinary surveillance systems. Integrating human and animal health interventions within a One Health framework will be critical to interrupt transmission, reduce long-term dependence on human PEP, and achieve Bangladesh’s “Zero by 30” rabies elimination goals.

While this study provides valuable insights, it has several limitations that warrant consideration. Although this study included all eligible patients presenting to the NRPCC during the study period, the relatively small number of compliant patients may have limited the statistical power to detect weaker associations between certain predictors and PEP compliance. Therefore, some potentially clinically relevant factors may not have reached statistical significance in the adjusted analysis. Being hospital-based, the findings may not represent individuals who never seek rabies PEP after an exposure, potentially underestimating noncompliance in the broader population. A small number of individuals recorded as WHO Category I received vaccines despite PEP not being indicated under WHO guidelines, likely reflecting precautionary clinical practice or inconsistencies in exposure categorization, and highlighting the need for continued provider education and guidelines adherence. The NRPCC database recorded exposure type as bite or scratch only and did not capture mucosal or other non-bite exposures recognized under WHO Category III criteria. Consequently, such exposures may have been underrepresented in our analysis, despite their recognized potential to result in rabies if appropriate PEP is not administered [37]. While IDH serves as a national referral center, it might attract a specific demographic that is already more motivated or able to travel for specialized care. Self-reported data on reasons for delay and adherence are susceptible to recall and social desirability biases. Because follow-up outcome data were not available in the secondary clinical records, the study could not evaluate subsequent rabies-related mortality or clinical outcomes associated with incomplete PEP compliance. While several associations were statistically significant, causal inferences cannot be definitively established due to the observational study design. Although our model showed good discrimination and acceptable calibration, the moderate sample size and categorization of continuous variables may have reduced model stability and generalizability, potentially causing information loss and threshold effects. Additionally, model performance may be optimistic because the threshold was derived from the same dataset without internal validation. Calibration was assessed using the Hosmer–Lemeshow test, but additional validation is needed to confirm model stability and generalizability. The six-month study period may not fully capture seasonal or longer-term temporal variations in healthcare-seeking behavior and PEP adherence, potentially limiting the ability to identify more stable or temporally distributed determinants of compliance. Future studies with extended observation periods and multicenter data are warranted to improve the robustness and generalizability of the findings.

## 5. Conclusions

This study highlights ongoing challenges in achieving full compliance with the WHO-recommended rabies PEP regimen among individuals presenting with animal exposures in Bangladesh, despite the provision of free services. Key determinants of adherence included socioeconomic status, gender, geographic accessibility, exposure severity, and initial wound-care practices. Consistent with evidence from other endemic settings, these findings underscore the need for targeted interventions to improve completion of the full WHO-recommended PEP regimen, including timely RIG administration for Category III exposures. Recommended strategies include decentralizing PEP delivery to reduce travel-related barriers, implementing reminder systems (e.g., SMS or phone calls) to minimize missed appointments, and developing tailored health education campaigns that emphasize the importance of completing the full PEP schedule for all WHO Category II and III exposures. Such efforts are essential to improving PEP uptake, reducing rabies mortality, and advancing Bangladesh’s goal of eliminating dog-mediated human rabies by 2030. The findings should be interpreted in light of several limitations, including the hospital-based single-center design, use of secondary data, and limited generalizability beyond patients attending the national referral center. Future initiatives should prioritize ongoing monitoring, impact evaluation, and qualitative research to better understand and address the behavioral and structural barriers contributing to non-compliance.

## Figures and Tables

**Table 1:** Summary statistics of key variables among patients with animal exposures who reported to National Rabies Prevention and Control Centre (NRPCC), Dhaka, Bangladesh (February–July 2023).

	Minimum	Maximum	Mean	SD
Age	1.00	70.00	25.88	15.60
Family income	10,000	450,000	34,105.03	40,661.61
Residence to IDH	1.00	110.00	10.42	9.40
Travel costs to the IDH	10.00	1000.00	147.98	113.14
Time gap between animal exposure to IDH visit	0.00	180.00	3.22	14.78
Medicine cost	150.00	800.00	344.73	45.97

**Table 2:** Determinants of adherence to the WHO-recommended rabies post-exposure prophylaxis (PEP) regimen among patients with animal exposures who reported to the National Rabies Prevention and Control Centre (NRPCC), Dhaka, Bangladesh (February–July 2023).

	Adhered to the WHO-Recommended PEP Regimen			
	Yes *n* (%)	No *n* (%)	Total *n* (%)	*p*-Value
Demographic characteristics				
Age category				
<15	14 (11.5)	108 (88.5)	122 (26.7)	0.055
≥15	64 (19.1)	271 (80.9)	335 (73.3)	
Gender				
Female	32 (22.5)	110 (77.5)	142 (31.1)	0.037
Male	46 (14.6)	269 (85.4)	315 (68.9)	
Profession				
Student	23 (14.1)	140 (85.9)	163 (35.7)	0.027
Job/Business	17 (12.9)	115 (87.1)	132 (28.9)	
Housewife	19 (23.2)	63 (76.8)	82 (17.9)	
Children	4 (13.3)	26 (86.7)	30 (6.6)	
Others	15 (30.0)	35 (70.0)	50 (10.9)	
Income Category				
<30,000	59 (28.4)	149 (71.6)	208 (45.5)	<0.001
≥30,000	19 (7.6)	230 (92.4)	249 (54.5)	
Education				
No education or below the primary	16 (29.6)	38 (70.4)	54 (11.8)	0.114
Primary	14 (13.7)	88 (86.3)	102 (22.3)	
Secondary	25 (16.1)	130 (83.9)	155 (33.9)	
Higher Secondary	10 (13.9)	62 (86.1)	72 (15.8)	
Above higher secondary	13 (17.6)	61 (82.4)	74 (16.2)	
Residence				
Dhaka North	42 (15.1)	178 (84.9)	279 (61.1)	0.303
Dhaka South	25 (19.2)	111 (80.8)	130 (28.4)	
Outside Dhaka	11 (22.9)	37 (77.1)	48 (10.5)	
Distance: Patient residence to IDH				
<10 (below avg)	55 (22.1)	194 (77.9)	249 (54.5)	0.002
≥10 (above avg)	23 (11.1)	185 (88.9)	208 (45.5)	
Know about IDH before				
Yes	46 (13.4)	297 (86.6)	343 (75.1)	<0.001
No	32 (28.1)	82 (71.9)	114 (24.9)	
Heard about IDH				
Doctor	9 (15.5)	49 (84.5)	58 (12.7)	<0.001
Neighbor	32 (12.2)	231 (87.8)	263 (57.5)	
Relative	25 (25.0)	75 (75.0)	100 (21.9)	
Others	12 (33.3)	24 (66.7)	36 (7.9)	
Characteristics of animals’ exposure				
Type of Animal				
Dog	51 (21.9)	182 (78.1)	233 (51.0)	0.020
Cat	26 (12.1)	189 (87.9)	215 (47.0)	
Others	1 (11.1)	8 (88.9)	9 (2.0)	
Exposure type				
Bite	56 (21.1)	209 (78.9)	265 (58.0)	0.008
Scratch	22 (11.5)	170 (88.5)	192 (42.0)	
Number of exposure				
Single	49 (14.8)	282 (85.2)	331 (72.4)	0.037
Multiple	29 (23.0)	97 (77.0)	126 (27.6)	
Type of animal				
Stray	51 (20.8)	194 (79.2)	245 (53.6)	0.055
Community own	8 (8.5)	86 (91.5)	94 (20.6)	
Own pet	18 (15.8)	96 (84.2)	114 (24.9)	
Wild	1 (25.0)	3 (75.0)	4 (0.9)	
Reason for exposure				
Provoked by patient	27 (14.2)	163 (85.8)	190 (41.6)	0.004
Provoked by animals	4 (6.2)	61 (93.8)	65 (14.2)	
Unprovoked	45 (22.5)	155 (77.5)	200 (43.8)	
WHO exposure Category				
Cat-I	3 (75.0)	1 (25.0)	4 (0.9)	0.007
Cat-II	24 (15.0)	136 (85.0)	160 (35.0)	
Cat-III	51 (17.4)	242 (82.6)	293 (64.1)	
Received RIG				
Yes	189 (78.8)	51 (21.3)	240 (52.5)	0.013
No	190 (87.6)	27 (12.4)	217 (47.5)	
Measures taken following animal exposure				
Wash with water only	12 (18.5)	53 (81.5)	65 (14.2)	<0.001
Wash with water and soap	48 (15.1)	270 (84.9)	318 (69.6)	
Did nothing	8 (13.6)	51 (86.4)	59 (12.9)	
Others	10 (66.7)	5 (33.3)	15 (3.3)	
Total	78 (17.1)	379 (82.93)	457 (100)	

**Table 3:** Multivariate analysis of sociodemographic and exposure-related factors associated with adherence to the WHO-recommended rabies post-exposure prophylaxis (PEP) regimen among patients with animal exposures who reported to the National Rabies Prevention and Control Centre (NRPCC), Dhaka, Bangladesh, 2023.

	Adhered to the WHO-Recommended PEP Regimen			
	COR (95% CI)	*p*-Value	AOR (95% CI)	*p*-Value
Demographic characteristics				
Age category				
<15	1.82 (0.98–3.39)	0.058	1.46 (0.51–4.15)	0.481
≥15	Reference		Reference	
Gender				
Female	0.59 (0.36–0.97)	0.038	0.63 (0.28–0.91)	0.025
Male	Reference		Reference	
Profession				
Student	0.90 (0.46–1.77)	0.759	0.73 (0.30–1.83)	0.506
Job/Business	1.84 (0.93–3.61)	0.078	0.85 (0.26–2.77)	0.788
Housewife	0.94 (0.30–2.93)	0.910	0.48 (0.08–2.95)	0.431
Children	2.61 (1.23–5.52)	0.012	1.12 (0.36–3.47)	0.847
Others	Reference		Reference	
Income Category				
<30,000	4.79 (2.75–8.36)	<0.001	4.18 (2.07–8.47)	<0.001
≥30,000	Reference		Reference	
Education				
No education or below the primary	2.61 (1.08–6.34)	0.034	2.01 (0.47–8.58)	0.348
Primary	0.99 (0.41–2.36)	0.980	0.90 (0.26–3.15)	0.870
Secondary	1.19 (0.54–2.64)	0.664	1.12 (0.41–3.06)	0.818
Higher Secondary	1.32 (0.54–3.24)	0.543	1.95 (0.62–6.11)	0.250
Above higher secondary	Reference		Reference	
Residence				
Dhaka North	1.34 (0.78–2.32)	0.289	2.25 (1.06–4.75)	0.034
Dhaka South	1.68 (0.79–3.55)	0.176	2.39 (1.11–5.38)	0.048
Outside Dhaka	Reference		Reference	
Distance: Patient residence to IDH				
<10	2.28 (1.35–3.86)	0.002	3.82 (1.64–8.93)	0.002
≥10	Reference		Reference	
Heard about IDH				
Doctor	0.37 (0.14–0.99)	0.048	0.53 (0.14–2.03)	0.356
Neighbor	0.28 (0.13–0.61)	<0.001	0.64 (0.22–1.84)	0.408
Relative	0.67 (0.29–1.53)	0.337	1.17 (0.39–3.50)	0.784
Others	Reference		Reference	
Know about IDH before				
Yes	0.40 (0.24–0.66)	<0.001	0.33 (0.17–0.65)	<0.001
No	Reference		Reference	
Characteristics of animals’ exposure				
Animal exposure				
Dog	0.49 (0.29–0.82)	0.007	0.59 (0.25–0.94)	0.022
Cat	0.45 (0.16–3.65)	0.452	0.29 (0.11–2.66)	0.274
Others	Reference		Reference	
Exposure type				
Scratch	2.07 (1.22–3.53)	0.007	1.77 (1.16–3.66)	0.012
Bite	Reference		Reference	
Number of exposures				
Single	0.58 (0.35–0.97)	0.038	0.41 (0.20–0.81)	0.010
Multiple	Reference		Reference	
Type of animal				
Stray	0.35 (0.16–0.78)	0.010	0.36 (0.13–0.95)	0.039
Community own	0.71 (0.40–1.29)	0.262	0.48 (0.20–1.18)	0.112
Own pet	1.27 (0.13–2.45)	0.084	1.90 (0.71–2.94)	0.175
Wild	Reference		Reference	
Reason for exposure				
Provoked by patient	0.40 (0.13–1.18)	0.096	0.46 (0.13–1.67)	0.236
Provoked by animals	1.75 (1.04–2.96)	0.036	1.49 (0.74–2.98)	0.265
Unprovoked	Reference		Reference	
WHO Category of Exposure				
Cat-I	2.24 (1.45–3.62)	0.023	3.48 (0.75–8.28)	0.074
Cat-II	0.84 (0.49–1.42)	0.511	0.81 (0.40–1.66)	0.573
Cat-III	Reference		Reference	
Receive RIG				
Yes	0.53 (0.32–0.88)	0.013		
No	Reference			
Measures taken following animal exposure				
Wash with water and soap	0.21 (0.13–0.49)	0.001	0.48 (0.17–1.98)	0.248
Wash with water only	0.19 (0.13–0.37)	<0.001	0.29 (0.14–0.91)	0.024
Others	0.18 (0.12–0.39)	<0.001	0.22 (0.12–0.84)	0.022
Did nothing	Reference		Reference	

COR = Crude Odds Ratio; AOR = Adjusted Odds Ratio (Reference category used in the logistic regression analysis against which other categories were compared for estimation of odds ratios).

**Table 4:** Model performance metrics including Hosmer–Lemeshow test, AUC, and classification accuracy.

Hosmer and Lemeshow Test			Area Under the Curve (AUC)		Classification Accuracy
Chi-Square	df	*p*-Value	Area	95% CI	
5.97	8	0.651	85.20%	83.96–87.51%	87.50%

## Data Availability

Data supporting the findings of this study can be found in the publication and its Supplementary Information Files.

## Supplementary Materials

The following supporting information can be downloaded at: https://www.mdpi.com/article/10.3390/tropicalmed11060165/s1.
